# Association between biomarkers of endothelial injury and hypocoagulability in patients with severe sepsis: a prospective study

**DOI:** 10.1186/s13054-015-0918-5

**Published:** 2015-04-24

**Authors:** Sisse Rye Ostrowski, Nicolai Haase, Rasmus Beier Müller, Morten Hylander Møller, Frank Christian Pott, Anders Perner, Pär Ingemar Johansson

**Affiliations:** Section for Transfusion Medicine, Capital Region Blood Bank, Copenhagen University Hospital, Rigshospitalet, Blegdamsvej 9, DK-2100 Copenhagen, Denmark; Department of Intensive Care, Copenhagen University Hospital, Rigshospitalet Blegdamsvej 9, DK-2100 Copenhagen, Denmark; Department of Intensive Care, Copenhagen University Hospital, Bispebjerg Hospital, Bispebjerg Bakke 23, DK-2400 Copenhagen, Denmark; Department of Surgery, Division of Acute Care Surgery, Centre for Translational Injury Research (CeTIR), University of Texas Medical School at Houston, 6410 Fannin Street UPB 1100, Houston, TX 77030 USA

## Abstract

**Introduction:**

Patients with severe sepsis often present with concurrent coagulopathy, microcirculatory failure and evidence of vascular endothelial activation and damage. Given the critical role of the endothelium in balancing hemostasis, we investigated single-point associations between whole blood coagulopathy by thrombelastography (TEG) and plasma/serum markers of endothelial activation and damage in patients with severe sepsis.

**Methods:**

A *post-hoc* multicenter prospective observational study in a subgroup of 184 patients from the Scandinavian Starch for Severe Sepsis/Septic Shock (6S) Trial. Study patients were admitted to two Danish intensive care units. Inclusion criteria were severe sepsis, pre-intervention whole blood TEG measurement and a plasma/serum research sample available from baseline (pre-intervention) for analysis of endothelial-derived biomarkers. Endothelial-derived biomarkers were measured in plasma/serum by enzyme-linked immunosorbent assay (syndecan-1, thrombomodulin, protein C (PC), tissue-type plasminogen activator and plasminogen activator inhibitor-1). Pre-intervention TEG, functional fibrinogen (FF) and laboratory and clinical data, including mortality, were retrieved from the trial database.

**Results:**

Most patients presented with septic shock (86%) and pulmonary (60%) or abdominal (30%) focus of infection. The median (IQR) age was 67 years (59 to 75), and 55% were males. The median SOFA and SAPS II scores were 8 (6 to 10) and 56 (41 to 68), respectively, with 7-, 28- and 90-day mortality rates being 21%, 39% and 53%, respectively. Pre-intervention (before treatment with different fluids), TEG reaction (R)-time, angle and maximum amplitude (MA) and FF MA all correlated with syndecan-1, thrombomodulin and PC levels. By multivariate linear regression analyses, higher syndecan-1 and lower PC were independently associated with TEG and FF hypocoagulability at the same time-point: 100 ng/ml higher syndecan-1 predicted 0.64 minutes higher R-time (SE 0.25), 1.78 mm lower TEG MA (SE 0.87) and 0.84 mm lower FF MA (SE 0.42; all *P* <0.05), and 10% lower protein C predicted 1.24 mm lower TEG MA (SE 0.31).

**Conclusions:**

In our cohort of patients with severe sepsis, higher circulating levels of biomarkers of mainly endothelial damage were independently associated with hypocoagulability assessed by TEG and FF. Endothelial damage is intimately linked to coagulopathy in severe sepsis.

**Trial registration:**

Clinicaltrials.gov number: NCT00962156. Registered 13 July 2009.

## Introduction

Sepsis coagulopathy evolves from initial activation of coagulation and fibrinolysis followed by late fibrinolytic shutdown and exhaustion of the natural anticoagulant systems [1], the latter mainly due to progressive endothelial disruption and damage [2-6]. Though patients with severe sepsis display evidence of varying degrees of coagulopathy with low or declining platelet count, low coagulation factor and fibrinogen levels and high or increasing D-dimer level, these conventional coagulation parameters do not reveal functional whole blood hemostasis, including changes in fibrinolysis and platelet (dys)function [2,6-10]. In contrast, viscoelastic hemostatic whole blood tests like thrombelastography (TEG®, Hemoscope-Hemonetics, Niles, IL, US) and rotation thromboelastometry (ROTEM®, TEM Inc., Durham, NC, US) [11] display functional hemostasis in whole blood, including fibrinolysis and platelet function, and multiple studies have characterized sepsis coagulopathy by these tests [12-22]. The endothelium plays a key role in balancing hemostasis [23-27], and there is emerging evidence that endothelial activation and damage are critical drivers of coagulopathy in acute critical illness, including severe sepsis [5,23-37].

Given that severe sepsis is associated with concurrent coagulopathy and endothelial disruption and damage, the aim of the present study was to investigate the association between coagulopathy, evaluated by whole blood TEG, and endothelial activation and/or damage in patients with severe sepsis. We hypothesized that high levels of biomarkers reflecting endothelial activation and/or damage would be independently linked to concurrent whole blood TEG hypocoagulability. In brief, the study confirmed our hypothesis by demonstrating that single time-point measurements of plasma markers of endothelial damage and, to a lesser degree, endothelial activation, were independently associated with concurrent whole blood hypocoagulability by TEG in patients with severe sepsis and septic shock. Clinically, endothelial protective interventions would be expected to attenuate the sepsis-induced hypocoagulability.

## Methods

### Study patients

The present study is a sub-study of The Scandinavian Starch for Severe Sepsis and Septic Shock (6S) trial [38], restricted to patients enrolled from March 2010 to November 2011 in two intensive care units (ICUs) at university hospitals in Denmark (Rigshospitalet, Bispebjerg Hospital). It was approved as a protocol amendment to the 6S trial by the Ethics Committee of the Capital Region of Denmark and the Danish Medicines Agency (Clinicaltrials.gov identifier: NCT00962156). Informed consent was obtained from all participants or their legal substitute prior to enrolment. The manuscript was prepared according to the Strengthening the Reporting of Observational studies in Epidemiology (STROBE) statement [39].

The 6S trial was a multicenter, blinded clinical trial in which ICU patients with severe sepsis were randomized to fluid resuscitation with either 6% hydroxyethyl starch (HES) 130/0.42 in Ringer’s acetate (Tetraspan 6%, B Braun, Melsungen, Germany) or Ringer’s acetate (Sterofundin ISO, B Braun, Melsungen, Germany). Eligible patients fulfilled the criteria for severe sepsis and needed fluid resuscitation as judged by the treating clinician. Exclusion criteria included renal replacement therapy (RRT), intracranial bleeding or infusion of over 1,000 ml of synthetic colloids at hospital admission, withdrawal and unobtainable consent [38].

Among the 798 patients in the intention-to-treat population [38], 226 patients were randomized at two ICUs that collected and stored research blood samples as part of the protocol after initiation of the present study (Rigshospitalet and Bispebjerg Hospital). Eligible patients were patients from these two ICUs who had concurrent pre-intervention (baseline) thrombelastography (TEG) measurement and endothelial biomarker measurements from baseline (data on both syndecan-1 and thrombomodulin as a minimum requirement), leaving 184 patients for analysis. Demography, disease severity and mortality were comparable between the excluded and included patients (data not shown).

Previously published data from the study include the primary results [38], as well as *post-hoc* analyses of the association between vasopressor therapy and endothelial damage in a subgroup of 67 patients randomized at two university hospitals in Denmark [40]. A further analysis of the association between consecutive TEG tracings and clinical outcome in a subgroup of 260 patients randomized at four university hospitals in Denmark has also been previously published [21].

### Collection of clinical data

The following data were retrieved from the 6S trial database: baseline patient demographics, co-morbidities, source of sepsis (some patients had more than one source of infection), organ failures, disease severity scores (Simplified Acute Physiology Score (SAPS) II and Sepsis-related Organ Failure Assessment (SOFA) score without the cerebral component), physiology and biochemistry, treatment with anti- and procoagulant drugs, fluid balances, transfusion with blood products, and use of renal replacement therapy (RRT) and mechanical ventilation. Follow-up data included date of discharge from the ICU and date of death censored at one year.

### Routine biochemistry and hematology

Routine biochemistry variables were analyzed in a DS/EN ISO 15189 standardized laboratory (hemoglobin, white blood cell and platelet counts, creatinine, blood urea nitrogen (BUN), bilirubin, sodium and potassium), including measurements of factor II-VII-X (FII-VII-X, Owren type PT, reported as coagulation activity in percentage [41]), plasma fibrinogen (p-fibrinogen) and D-dimer (ACL TOP). Arterial blood gasses (ABG) and lactate were analyzed by an ABL 725 (Radiometer, Copenhagen, Denmark).

### Thrombelastography and functional fibrinogen

Standard TEG (kaolin citrated TEG) and functional fibrinogen (tissue factor citrated FF) were performed at time of inclusion (pre-randomization) in 3.2% citrated whole blood using a TEG® 5000 Hemostasis Analyzer System (Haemonetics Corp., MA, US). At both hospitals, TEG and FF analyses were performed at the blood bank, according to the manufacturer’s recommendations.

The TEG variables recorded were (normal range reported by Haemonetics Corp.): reaction time (R (3 to 9 minutes), rate of initial fibrin formation), angle (α (55 to 78 degrees), clot growth kinetics, reflecting clot growth and the thrombin burst), TEG maximum amplitude (TEG MA (51 to 69 mm), reflecting maximum clot strength) and lysis after 30 minutes (Ly30 (0 to 8%), proportional reduction in the amplitude 30 minutes after MA, reflecting fibrinolysis) [11]. The FF analysis included a platelet glycoprotein IIb/IIIa receptor inhibitor. FF was performed to investigate the contribution from fibrinogen to TEG MA (FF MA 14 to 27 mm).

The results of the TEG analyses were not made available for the treating clinicians, but TEG analyses were routinely used in the treatment of bleeding patients in the participating ICUs. Consequently, non-study TEG analyses and interventions with plasma, platelets and hemostatic agents based on non-study TEG results may have occurred in some patients. The day-to-day coefficient of variation percentage of TEG MA is less than 7% in our laboratory [42].

### Enzyme-linked immunosorbent assay

Soluble endothelial-derived biomarkers were measured by commercially available immunoassays in serum/plasma, sampled pre-intervention (baseline), according to the manufactures recommendations: syndecan-1 (Diaclone SAS, Besancon, France; lower limit of detection (LLD) 4.94 ng/ml) and soluble thrombomodulin (sTM, Nordic Biosite, Copenhagen, Denmark; LLD 0.31 ng/ml), both in serum; protein C (PC, Helena Laboratories, Beaumont, TX, US; relative quantification); tissue-type plasminogen activator (tPA, Americal Diagnostics (ADI), detects single-chain (sc)-tissue type plasminogen activator (tPA), two-chain (tc)-tPA and tPA/PAI-1 complexes; LLD 1 ng/ml) and plasminogen activator inhibitor-1 (PAI-1, Assaypro; LLD 0.07 ng/ml), all in citrated plasma. The biomarkers measured were selected to reflect distinct aspects of endothelial activation and damage: glycocalyx damage (syndecan-1 [43]), endothelial cell damage (soluble thrombomodulin (sTM) [44-46]), endothelial cell activation (tPA and PAI-1 [47]) and protein C-induced natural anticoagulation [48].

### Statistics

Statistical analysis was performed using SAS 9.1.3 SP4 (SAS Institute Inc., Cary, NC, US).Simple correlations were investigated by Pearson correlations, with results displayed as Pearson’s r, Pearson’s r^2^ (r^2^) and *P* values. To investigate the independent association between endothelial activation and damage (syndecan-1, thrombomodulin, protein C, tPA and PAI-1) and coagulopathy evaluated by TEG whole blood hemostasis (R-time, angle, TEG MA and FF MA), univariate and backwards multivariate linear regression analysis were performed, including variables that were either found to correlate with or expected to influence TEG variables: SOFA score (encompasses assessment of the ratio of partial pressure arterial oxygen and fraction of inspired oxygen (PaO_2_/FiO_2_), mean arterial blood pressure (MAP)/vasopressor requirement, bilirubin, platelet count and creatinine), D-dimer, Owren type PT, p-fibrinogen and crystalloid administration (the previous 24 hours before blood sampling). Individual backwards multivariate linear regression models, including only univariate significant variables, were applied for each investigated biomarker (syndecan-1, thrombomodulin and protein C). Results are presented as regression coefficients (β) with 95% confidence intervals (CI) and t and *P* values. The association between syndecan-1 quartile and TEG and FF variables were investigated by univariate linear regression analysis, with syndecan-1 quartile as the explanatory variable, with results displayed as regression coefficients (β) with 95% CIs and *P* values. The association between syndecan-1 quartile and presence of fibrinolysis (Ly30 > 0%, yes or no) was investigated by the Cochran-Armitage Trend Test, with results displayed as *P* values. Descriptive data are presented as medians with interquartile ranges (IQR) or as n (proportions). *P* values <0.05 were considered to be significant.

## Results

### Patients

The 184 patients included in the present study were randomized at two different trial sites (n = 140 (76%) at Rigshospitalet, n = 44 (24%) at Bispebjerg Hospital). The patients had a median age of 67 years, with 55% being male (Table 1). The majority of patients presented with pulmonary (60%) or abdominal (30%) focus of infection and had septic shock (n = 156 (86%)). A total of 34% of patients underwent emergency surgery before admission and 20% had hematologic malignancy (Table 1). The median SOFA and SAPS II scores at inclusion were 8 and 56, respectively, and 7-day, 28-day, 90-day and 1-year mortality rates were 21%, 39%, 53% and 61%, respectively (Table 1). Data on endothelial-derived biomarkers and TEG and FF are displayed in Table 1. Among the 184 patients, 23 (13%), 90 (49%) and 71 (39%) presented with a hypocoagulable, normal and hypercoagulable TEG MA, respectively, whereas 35 (19%), 94 (51%) and 55 (30%) presented with a hypocoagulable, normal and hypercoagulable FF MA. A comparable number of patients were randomized into the two fluid intervention groups: 89 (48%) were allocated to Ringer’s acetate and 95 (52%) to HES 130/0.42.

**Table 1:** **Baseline (inclusion) characteristics, endothelial-derived biomarker levels, thrombelastography, transfusions during ICU stay and outcome in 184 patients with severe sepsis**

		**All**
**Variable**		**Median (IQR)**
Age	years	67 (59-75)
Male gender	n (%)	101 (55%)
**Disease severity**		
Lactate	mmol/l	2.2 (1.4-3.7)
SBP	mmHg	82 (70-92)
SOFA	score	8 (6-10)
SAPS	score	56 (41-68)
**Biochemistry**		
Hemoglobin	g/dl	9.5 (8.4-10.8)
White blood cells	10^9^/l	12.3 (7.4-20.1)
Platelet count	10^9^/l	133 (71-232)
Creatinine	μmol/l	97 (62-172)
BUN	μmol/l	10.5 (6.8-17.7)
Bilirubin	μmol/l	12 (7-21)
D-dimer	mg FEU/l	4.8 (2.2-9.6)
Owren type PT	%	0.47 (0.34-0.61)
Plasma fibrinogen	g/l	4.7 (3.4-6.5)
**Endothelial biomarkers**		
Syndecan-1	ng/ml	97 (51-204)
Thrombomodulin	ng/ml	7.7 (5.2-10.8)
Protein C	%	47.4 (33.5-66.4)
tPA	ng/ml	16.4 (11.4-23.6)
PAI	ng/ml	30.9 (19.3-39.5)
**Thrombelastography (TEG)**		
R-time	min	7.7 (6.4-9.3)
Angle	degrees	65 (55-71)
TEG MA	mm	66 (58-72)
Ly30	%	0 (0.0-0.3)
Ly30 > 0%	n (%)	65 (36%)
**Functional fibrinogen (FF)**		
FF MA	mm	21.9 (16.1-28.8)
**Transfusions in ICU**		
RBC	n (%)	119 (65%)
FFP	n (%)	64 (35%)
Platelets	n (%)	52 (28%)
**Mortality**		
7-day	n (%)	39 (21%)
28-day	n (%)	72 (39%)
90-day	n (%)	98 (53%)
1-year	n (%)	113 (61%)

Data are presented as medians (IQR) or n (%). BUN, blood urea nitrogen; Emergency surgery, emergency surgery prior to ICU admission; FF MA, maximum amplitude in functional fibrinogen; FEU, fibrinogen equivalent units; FFP, fresh frozen plasma; Ly30, lysis 30 minutes after MA; Mortality; mortality of any cause; Owren type PT, reporting coagulation factor activity in percentage [41]; PAI-1, plasminogen activator inhibitor-1; R-time, reaction time; RBC, red blood cells; SAPS, simplified acute physiology score (calculated from 17 variables with higher scores indicating more severe disease, but without recording the cerebral component of the score); SBP, systolic blood pressure; SOFA, sequential organ failure assessment (sub-scores ranging from 0 to 4 for each of five components (circulation, lungs, liver, kidneys and coagulation, with aggregated scores ranging from 0 to 20, with higher scores indicating more severe organ failure); TEG MA, maximum amplitude in kaolin TEG; tPA, tissue-type plasminogen activator.

### Clinical presentation and endothelial damage

Neither focus of infection nor presence of shock or emergency surgery before admission influenced syndecan-1, thrombomodulin or protein C quartiles (data not shown). Patients with hematologic malignancy, however, were in a high thrombomodulin quartile (*P* = 0.036), and patients with shock at admission were in a higher tPA and PAI-1 quartile (both *P* <0.01, data not shown).

### Correlations and syndecan-1 quartiles

The TEG R-time, angle and MA all correlated weakly with SOFA score (r = 0.27 (r^2^ 0.07), r = −0.30 (r^2^ 0.09), r = −0.34 (r^2^ 0.12), respectively, all *P* <0.001), lactate (r = 0.18 (r^2^ 0.03), r = −0.25 (r^2^ 0.06), r = −0.32 (r^2^ 0.11), respectively, all *P* <0.05), platelet count (r = −0.35 (r^2^ 0.13), r = 0.57 (r^2^ 0.32), r = 0.69 (r^2^ 0.48), respectively, all *P* <0.001), D-dimer (r^2^ 0.02, r^2^ 0.03 and r^2^ 0.04, respectively, Table 2, univariate model) and Owren type PT (r^2^ 0.03, r^2^ 0.03 and r^2^ 0.07, respectively Table 2, univariate model); TEG angle and MA correlated with p-fibrinogen (r^2^ 0.05 and r^2^ 0.23, respectively, Table 2, univariate model). Also, FF MA correlated with platelet count (r^2^ 0.07), D-dimer (r^2^ 0.06), Owren type PT (r^2^ 0.17), p-fibrinogen (r^2^ 0.67) and crystalloids administered in the 24 hours prior to blood sampling and randomization (r^2^ 0.03) (all Table 2, univariate model). TEG Ly30 correlated with SOFA score (r = 0.15 (r^2^ 0.02), *P* = 0.044) and lactate (r = −0.42 (r^2^ 0.18), *P* <0.001). Transfusions administered the in the 24 hours prior to blood sampling and randomization neither correlated with TEG and FF nor with endothelial biomarkers (data not shown). TEG and FF variables all correlated with syndecan-1, thrombomodulin and protein C (Table 2, univariate model). Furthermore, PAI-1 correlated with TEG MA (r = −0.20 (r^2^ 0.04), *P* = 0.006) and FF MA (r = −0.37 (r^2^ 0.13), *P* <0.001). Also, increasing disseminated intravascular coagulation (DIC) score was associated with increased syndecan-1 (*P* <0.001) and thrombomodulin (*P* <0.001) and reduced protein C (*P* <0.001), whereas tPA and PAI-1 remained stable across DIC scores (data not shown).

**Table 2:** **Univariate and backwards multivariate linear regression analyses of associations between coagulopathy evaluated by TEG and biomarkers of endothelial activation and damage in 184 patients with severe sepsis**

**Variable**	**Unit**	**TEG R-time**			**TEG Angle**			**TEG MA**			**FF MA**		
		**β (SE)**	**t**	***P***	**β (SE)**	**t**	***P***	**β (SE)**	**t**	***P***	**β (SE)**	**t**	***P***
**Univariate**													
Syndecan-1	100 ng/ml	0.82 (0.24)	3	**0.001**	−3.06 (091)	3	**0.001**	−3.40 (0.79)	−4	**<0.001**	−2.00 (0.64)	−3	**0.002**
Thrombomodulin	ng/ml	0.13 (0.06)	2	**0.021**	−0.49 (0.22)	−2	**0.026**	−0.60 (0.19)	−3	**0.002**	−0.58 (0.15)	−4	**<0.001**
Protein C	10%	−0.20 (0.10)	−2	**0.044**	1.09 (0.37)	3	**0.004**	1.49 (0.32)	5	**<0.001**	1.05 (0.25)	4	**<0.001**
SOFA score	point	0.30 (0.08)	4	**<0.001**	−1.25 (0.30)	−4	**<0.0001**	−1.27 (0.26)	−5	**<0.001**	-	-	NS
D-dimer	mg FEU/l	0.03 (0.02)	2	**0.044**	−0.13 (0.06)	−2	**0.026**	−0.13 (0.05)	−3	**0.011**	−0.14 (0.04)	−4	**0.001**
Owren type PT	10%	−0.29 (0.12)	−2	**0.020**	1.02 (0.46)	2	**0.029**	1.39 (0.40)	4	**0.001**	1.80 (0.30)	6	**<0.001**
P-fibrinogen	g/l	-	-	NS	1.41 (0.47)	3	**0.003**	2.62 (0.35)	7	**<0.001**	3.50 (0.18)	19	**<0.001**
Crystalloid	100 ml	-	-	NS	-	-	NS	-	-	NS	−0.10 (0.04)	−2	**0.022**
**Multivariate**													
Syndecan-1	100 ng/ml	**0.64 (0.25)**	**3**	**0.013**	-	-	NS	**−1.78 (0.87)**	**−2**	**0.042**	**−0.84 (0.42)**	**−2**	**0.045**
SOFA score	point	0.23 (0.08)	3	0.006	−1.19 (0.30)	−4	<0.001	−1.07 (0.27)	−4	<0.001	Not included		
D-dimer	mg FEU/l	-	-	NS	-	-	NS	-	-	NS	-	-	NS
Owren type PT	10%	-	-	NS	-	-	NS	0.96 (0.40)	2	0.020	0.86 (0.23)	3	0.001
P-fibrinogen	g/l	Not included			1.26 (0.44)	3	0.004	-	-	NS	3.44 (0.21)	17	<0.001
Crystalloid	100 ml	Not included			Not included			Not included			-	-	NS
**Multivariate**													
Thrombomodulin	ng/ml	-	-	NS	-	-	NS	-	-	NS	-	-	NS
SOFA score	point	0.28 (0.08)	2	<0.001	−1.19 (0.30)	−4	<0.001	−1.24 (0.26)	−5	<0.001	Not included		
D-dimer	mg FEU/l	-	-	NS	-	-	NS	-	-	NS	-	-	NS
Owren type PT	10%	−0.26 (0.12)	−2	0.031	-	-	NS	1.27 (0.38)	3	<0.001	0.54 (0.20)	3	0.007
P-fibrinogen	g/l	Not included			1.26 (0.44)	3	0.004	-	-	NS	3.35 (0.21)	17	<0.001
Crystalloid	100 ml	Not included			Not included			Not included			-	-	NS
**Multivariate**													
Protein C	10%	-	-	NS	-	-	NS	**1.24 (0.31)**	**4**	**<0.001**	-	-	NS
SOFA score	point	0.28 (0.08)	4	<0.001	−1.19 (0.30)	−4	<0.001	−1.09 (0.26)	−4	<0.001	Not included		
D-dimer	mg FEU/l	-	-	NS	-	-	NS	-	-	NS	-	-	NS
Owren type PT	10%	−0.26 (0.19)	−2	0.031	-	-	NS	-	-	NS	0.54 (0.20)	3	0.007
P-fibrinogen	g/l	Not included			1.26 (0.44)	3	0.004	-	-	NS	3.35 (0.21)	17	<0.001
Crystalloid	100 ml	Not included			Not included			Not included			-	-	NS

Regression coefficients (β) with standard errors (SE), t and *P* values displayed for the univariate and backwards selection multivariate models. Individual backwards multivariate linear regression models including only univariate significant variables, were applied for each investigated biomarker (syndecan-1, sTM and protein C). *P* values are shown in bold for endothelial-derived biomarkers with *P* <0.05. Predicted change in TEG R-time (minutes), TEG angle (degrees),TEG MA (maximum amplitude, mm) or FF MA (maximum amplitude, mm) associated with one unit increase in syndecan-1 (100 ng/ml), thrombomodulin (ng/ml), protein C (10%), SOFA score (point), D-dimer (mg FEU/l), Owren type PT (10%), plasma (p)-fibrinogen (g/l) and crystalloids administered in the 24 hours prior to blood sampling and randomization (100 ml).

**Figure 1:**
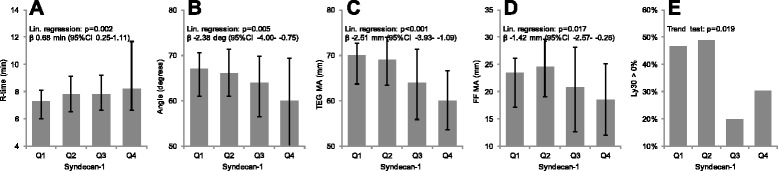
Thrombelastography (TEG) and functional fibrinogen (FF) variables in 184 patients with severe sepsis stratified according to plasma syndecan-1 quartiles. The median and interquartile ranges **(A-D)** or proportions **(C)** are shown for: **A)** TEG reaction time (R-time; minutes), **B)** TEG angle (degrees), **C)** TEG maximum clot strength (MA; mm), **D)** FF MA (mm) and **E)** Ly30 > 0% (proportion). The influence of syndecan-1 quartile in Figure 1A-D on TEG and FF variables were investigated by linear regression analysis with syndecan-1 quartile as the explanatory variable, with results displayed as regression coefficients (β) with 95% confidence intervals and *P* values. Presence of fibrinolysis (Ly30 > 0%) across syndecan-1 quartiles in Figure 1E was investigated by Cochran-Armitage Trend Test, with results displayed as *P* values.

In accordance with the correlations, higher syndecan-1 quartile was associated with TEG and FF hypocoagulability, evidenced by increased R-time and reduced angle, TEG and FF MA (Figure 1A-D), whereas it was associated with a reduced proportion of patients presenting with TEG fibrinolysis (Figure 1E).

### Linear regression analysis of the association between whole blood coagulopathy and endothelial activation and damage

The independent association at one time-point between coagulopathy and endothelial activation and damage was investigated by linear regression with individual backwards multivariate linear regression models, including only univariate significant variable applied for each investigated biomarker (syndecan-1, thrombomodulin and protein C) (Table 2).

In the univariate analyses, higher SOFA score and D-dimer, but lower Owren type PT and p-fibrinogen, were associated overall with more hypocoagulable TEG and FF profiles, corresponding to increased R-time and reduced angle, TEG MA and TEG FF (Table 2). Similarly, higher syndecan-1 and sTM and lower protein C were associated with more hypocoagulable TEG and FF profiles in the univariate analyses. Higher PAI-1 was associated with a more hypocoagulable TEG MA (β: −0.20 mm (95% CI: −0.34 to −0.06), *P* = 0.006) and FF MA (β: −0.29 mm (95% CI: −0.40 to −0.18), *P* <0.001), whereas tPA was neither associated with TEG nor FF variables (data not shown).

In the multivariate analyses including the univariate significant variables and either syndecan-1, thrombomodulin or protein C, higher syndecan-1 was independently associated with TEG and FF hypocoagulability, evidenced by increased R-time and reduced TEG MA and FF MA (Table 2). Also, lower protein C was associated with reduced TEG MA (Table 2), whereas PAI-1 and tPA were not independently associated with TEG or FF MA (data not shown). In addition to syndecan-1 and protein C, higher SOFA score was consistently independently associated with TEG and FF hypocoagulability (Table 2), whereas lower Owren type PT and p-fibrinogen were independently associated with TEG and FF hypocoagulability in some multivariate analyses (Table 2).

## Discussion

The main finding of the present study was that plasma markers of endothelial damage and, to a lesser degree, endothelial activation, were independently associated with concurrent whole blood hypocoagulability by TEG in patients with severe sepsis and septic shock. Furthermore, fibrinolysis was inversely associated with syndecan-1 quartiles, suggesting a negative influence of glycocalyx damage on fibrinolysis.

The vascular endothelium influences hemostasis, locally or systemically, through controlled release, expression and/or support of systems and/or elements that either promote or inhibit coagulation [23-27]. There is emerging evidence that the endothelium, actively or passively, contributes to coagulopathy in acute critical illness, including sepsis [5,23-26,28-37,49]. The endothelium comprises a single layer of cells that lines the inner of all blood vessels and traverses all organs in the body, establishing a unique interface between the underlying tissue and flowing blood [25]. The luminal surface of the endothelial cells is covered by the glycocalyx that binds and incorporates soluble molecules derived from the plasma and endothelium, with the highest amounts of plasma-derived constituents towards the luminal surface [50]. It provides the endothelium with an anti-adhesive and anticoagulant surface that protects the underlying endothelial cells and contributes to control of vascular barrier function [51-53].

Glycocalyx damage, evidenced by increased levels of circulating syndecan-1 [43], can range from discrete disturbances in the composition of the most luminal layer, to excessive destruction and degradation, with loss of the entire glycocalyx [50,51]. Clinically, glycocalyx and endothelial cell damage are associated with pathophysiologic sequels like capillary leakage and tissue edema, accelerated inflammation and platelet activation, microvascular thrombus formation, loss of vascular responsiveness, hypotension, microcirculatory collapse and (multiple) organ failure [6,51,52,54,55].

Importantly, upon shedding, the glycocalyx constituents retain their anticoagulant heparin-like activity [49] and promote measurable TEG hypocoagulability in the flowing blood through endogenous heparinization, as demonstrated in hepatic failure, severe sepsis [31,32,49] and trauma [35]. This may mechanistically explain part of the association between syndecan-1 and TEG hypocoagulability observed in the present study. Also, the association between lower protein C levels and TEG hypocoagulability may be attributed to enhanced protein C activation, and consequently increased endogenous anticoagulation, due to inactivation of factors V and VIII and induction of hyperfibrinolysis [56-59]. Together, these findings suggest that endothelial damage, and to a lesser degree activation, may be linked to functional whole blood hypocoagulability in severe sepsis. Clinically, endothelial protective interventions would hereby be expected to attenuate the sepsis-induced hypocoagulability. However, given the relatively weak correlations between endothelial activation and damage markers and TEG/FF variables, as evaluated by the coefficients of determination (r^2^), it should be emphasized that factors besides the endothelium may also contribute significantly to the observed hypocoagulability. Also, the observational nature of the study makes it impossible to establish causality, meaning that the association between endothelial damage and TEG hypocoagulability could also be attributed to coagulation factor and platelet consumption due to microthrombi formation.

Acute critically ill patients suffering from non-septic disease, such as severe trauma and resuscitated cardiac arrest, also display evidence of excessive endothelial activation and damage concurrent with coagulopathy [28,29,60-67]. We recently proposed that the coagulopathy observed in severe trauma and other types of acute critical illness reflects a universal response mounted to counterbalance emerging systemic microvascular and endothelial disruption and damage inflicted by the ‘injurious hit’, that is, the tissue injury, infection and/or ischemia-reperfusion injury and its concurrent activation of the neurohumoral, inflammatory and hemostatic systems [37]. Recent studies of trauma, sepsis and burn patients support the notion that different injurious hits generate a common genetic response [68,69]. Consequently, we infer that the coagulopathy observed in acute critically ill and shocked patients may reflect that evolution has prioritized a response that ensures oxygen supply above hemostasis in these patients [37].

In the present study, excessive glycocalyx damage (evidenced indirectly by high circulating syndecan-1 levels) was inversely associated with functional TEG hyperfibrinolysis. Though excessive hyperfibrinolysis represents an extreme form of coagulopathy observed in some critically ill and shocked patients suffering from, for example, severe trauma [70-75], resuscitated cardiac arrest [36,76-78] and septic shock [15], the ‘fibrinolytic shutdown’ and enhanced thrombus generation in severe sepsis may limit pathogen dissemination but drive organ failure [9,10,79-81]. Thus, the inverse correlation between Ly30 and syndecan-1 may suggest that ‘physiologic’ fibrinolysis is an active endothelial process that may be impaired by excessive endothelial activation and glycocalyx degradation.

The results presented here are subject to several limitations. First, the observational nature of the study does not allow independent evaluation of the cause-and-effect relationship suggested, as mentioned above. Second, as this was a sub-study to the 6S trial, some of the exclusion criteria may not be entirely relevant for the present study, which may reduce the generalizability of the results. Third, we did not adjust for multiple testing, emphasizing that results obtained from a *post-hoc* subgroup study such as in this study, should be interpreted with caution and optimally confirmed in later studies designed specifically for that purpose. Fourth, the data presented here were based on single time-point measurements and not consecutive data. To establish the potential causality and improve our understanding of the interaction between the two extremely complex biologic systems (the endothelium and hemostasis) in patients with complex diseases like sepsis, prospective randomized studies are warranted, investigating hemostasis in patients treated with intervention(s) expected to protect the endothelium.

## Conclusions

We found that a single time-point measurement of plasma markers of endothelial damage and, to a lesser degree, endothelial activation, were independently associated with whole blood hypocoagulability by TEG in patients with severe sepsis, and that fibrinolysis correlated inversely with markers of glycocalyx damage. These findings suggest that endothelial activation and damage may be linked to hypocoagulability in patients with severe sepsis.

## Key messages

- A single time-point measurement of plasma markers of endothelial damage and, to a lesser degree, endothelial activation, were independently associated with whole blood hypocoagulability by TEG in patients with severe sepsis and septic shock.
- Fibrinolysis was inversely associated with syndecan-1, a marker of glycocalyx damage.
- These findings suggest that endothelial activation and damage may be linked to hypocoagulability in patients with severe sepsis.
- Clinically, endothelial protective interventions would be expected to attenuate sepsis-induced hypocoagulability.

## Abbreviations

6S trial: The Scandinavian Starch for Severe Sepsis and Septic Shock trial
ABG: Arterial blood gasses
DIC: Disseminated intravascular coagulation
ELISA: Enzyme-linked immunosorbent assay
FF: Functional fibrinogen
HES: Hydroxyethyl starch
ICU: Intensive care unit
IQR: Interquartile range
Ly30: Proportional reduction in the amplitude 30 minutes after MA, reflecting fibrinolysis
MA: Maximum amplitude
MAP: Mean arterial blood pressure
PAI-1: Plasminogen activator inhibitor-1
ROTEM: Rotation thromboelastometry
RRT: Renal replacement therapy
R-time: Reaction time
SAPS score: Simplified Acute Physiology Score
SOFA score: Sepsis-related Organ Failure Assessment (SOFA) score
sTM: Soluble thrombomodulin
STROBE: the Strengthening the Reporting of Observational Studies in Epidemiology
TEG: Thrombelastography
tPA: Tissue-type plasminogen activator
α: Angle
